# Ultrathin-Layer Structure of BiOI Microspheres Decorated on N-Doped Biochar With Efficient Photocatalytic Activity

**DOI:** 10.3389/fchem.2019.00378

**Published:** 2019-05-24

**Authors:** Jianhua Hou, Ting Jiang, Rui Wei, Faryal Idrees, Detlef Bahnemann

**Affiliations:** ^1^School of Environmental Science and Engineering, Yangzhou University, Yangzhou, China; ^2^Jiangsu Collaborative Innovation Center for Solid Organic Waste Resource Utilization, Nanjing, China; ^3^Department of Photocatalysis and Nanotechnology, Institut für Technische Chemie, Gottfried Wilhelm Leibniz Universität Hannover, Hannover, Germany; ^4^Department of Physics, The University of Lahore, Lahore, Pakistan; ^5^Laboratorium für Nano- und Quantenengineering, Gottfried Wilhelm Leibniz Universität Hannover, Hannover, Germany

**Keywords:** BiOI, microsphere, ultrathin nanosheets, *in-situ*, self-template, visible-light photocatalysis

## Abstract

Bismuth oxyiodide (BiOI) is among the most potential photocatalysts due to its photocatalytic activity under visible light irradiation. However, the photoinduced carrier separation efficiency has limited the BiOI photocatalytic activity. Herein, we utilized the direct carbonation of sapless cattail grass to obtain N-doped hierarchical structure cattail-based carbon (NCC). The NCC not only served as an appropriate host but also as a self-sacrificing template for BiOI microspheres for the preparation of BiOI/NCC composite material. The acidic solutions (HCl or AcOH) were used as a solvent which helped to obtain a well-defined micro/nano hierarchical BiOI microspheres composed of ultrathin nanosheets. Thus, BiOI/NCC composites were successfully designed through the *in-situ* self-template rapid dissolution-recrystallization mechanism. Additionally, numerous well-contacted interfaces were formed between NCC and BiOI, which served as an electron-acceptor bridge function for ultrafast electron transfer process in order to hinder the electron-hole pairs recombination. On account of the multiple synergistic effects of micro/nano hierarchical microsphere structure, ultrathin nanosheets, and well-contacted interface, the as-prepared BiOI/NCC composites exhibit the superior degradation of rhodamine B (RhB) than pure BiOI under visible light irradiation.

## Introduction

Photocatalysis technology is believed to be a green technology with great potential to thoroughly degrade refractory organic pollutants into non-toxic or low-toxic small molecules by using semiconductor materials as photocatalysts (Hoffmann et al., 1995; Jin et al., 2017). In recent years, BiOI has shown remarkable potential in visible-light photocatalysis owing to its special layered structure and suitable energy bandgap (Zhang et al., 2008; Jin et al., 2017; Liu G. et al., 2017). Unfortunately, due to the small lifetime of electron-hole pairs, the quantum efficiency of BiOI is very low. Therefore, its practical applications are very few (Dash et al., 2014; Hoye et al., 2017).

Several strategies have been adopted to overcome these shortcomings, such as doping (Dash et al., 2014), heterogeneous junctions (Huang et al., 2016; Wang Y. et al., 2018), crystal-facet control (Ye et al., 2016), morphology control (Xin et al., 2014; Huang et al., 2016; Jiang et al., 2017), and the deposition of noble metals (Hsu et al., 2018), etc. Among these, morphology control by adjusting the synthesis conditions have received extensive attention considering the possible photocatalytic reactions took place over the surface of semiconductor materials. On one hand, previous studies have shown that BiOI self-assembled into 3D hierarchical structures by 2D nanosheets can promote the efficient separation of photogenerated charge carriers (Lei et al., 2010; Hu et al., 2014; Huang et al., 2016). For example, Lei et al. (2010) prepared 3D hierarchical flower-shaped BiOI by a solvent method, which improved the catalytic activity and had a good catalytic effect. On the other hand, the ultrathin-layer formation is recognized as a promising new method, mainly because it enables the photoinduced charge carriers to generate faster transmission to reach the surface of BiOI, thus leading to superior separation of photoinduced electrons and holes in semiconductor (Zhang et al., 2014; Jiang et al., 2017). Noticeably, ultrathin-layer can extend the bandgap of BiOI, which indicates stronger oxidization ability (Jiang et al., 2017). For example, Jiang et al. (2017) synthesized hollow flower-like BiOI semiconductor (h-BiOI) with ultrathin nanosheets (the thickness is around 2 nm) using a solvothermal method, which displays improved activity in visible light irradiation. In addition, to further enhance the photocatalytic activity, it is an excellent strategy to use carbonaceous materials as the carrier for semiconductor materials to quickly capture electrons, thus slow down the recombination of electron-hole species (Gao et al., 2012; Di et al., 2018; Lu et al., 2018). Therefore, N-doped carbon-based composites reveal the advantages of good conductivity, and adjusted surface performance, etc., which are conducive to the super-fast electron transfer process through interfacial interaction (Hou et al., 2015, 2017; Yin et al., 2019). For example, Di et al. (2018) fabricated controllable N-doped carbon quantum dots (N-CQDs) with modified BiOI nanosheets nano-junctions, that improve the photocatalytic activity. Therefore, it is still a challenge to construct the optimized structure with ultrathin-layer in BiOI/N-doped carbon composites via a one-step, facile and green preparation method.

Our previous work has successfully fabricated lantern-like Bi_7_O_9_I_3_/bamboo tube-like carbon nanocomposites via buffer effect of NH_3_•H_2_O solution, and the catalyst has shown the superior photocatalytic performance (Hou et al., 2018). In this work, we utilized direct carbonation of biowaste (sapless cattail grass) in the ammonia atmosphere to obtain N-doped cattail-based carbon (NCC). Then using acidic solutions (HCl or AcOH) as solvent and NCC as a carrier, micro/nano hierarchical BiOI/NCC composites have been successfully designed via *in-situ* self-template rapid dissolution-recrystallization mechanism through a one-step approach. The even-distributed BiOI microspheres are composed of ultrathin nanosheets on the surface and inside of N-doped hierarchical structure carbon. Furthermore, numerous well-contacted interfaces can be formed between NCC and BiOI. Due to the multiple synergistic effects of the micro/nano hierarchical structure, ultrathin nanosheets and well-contacted interface, the as-prepared BiOI/NCC composites exhibited the superior photocatalytic activity of RhB than pure BiOI under visible light. Therefore, we proposed that controllable design self-assembled BiOI microspheres with ultrathin-layer structure loaded on biowaste-derived N-doped biochar is an efficient photocatalyst.

## Experimental Methods

### Preparation

The N-doped hierarchical cattail-based carbon (named NCC) was obtained from direct carbonation of biological waste (sapless cattail grass) in ammonia gas at 750°C for 3 h.

BiOI/NCC samples were produced by the following procedure: 60 mg of NCC was put into a round bottom bottle with a mixture of 50 mL ethylene glycol (EG) and 50 mL distilled water during continuous magnetic stirring, 10 mmol Bi(NO_3_)_3_·5H_2_O was then added in the reaction system. When Bi(NO_3_)_3_·5H_2_O get dissolved, 15 mL of thiourea (TU, 1 mol L^−1^) was added and stirred for 5 min, followed by 10 mmol KI with constant stirring for 30 min. Next, 100 mL hydrochloric acid solution (HCl, 12 mmol L^−1^) or 100 mL acetic acid solution (AcOH, 175 mmol L^−1^) was added dropwise. In the end, 200 mL of distilled water was added and stirred continuously for 1 h. The solid product is collected through vacuum filtration with washing by distilled water and ethanol and then was dried at 60°C for 12 h. Finally, the products obtained from the treatment of HCl and AcOH solutions were named BiOI/NCC-HCl and BiOI/NCC-AcOH, respectively. BiOI-HCl and BiOI-AcOH samples were prepared by a similar method without NCC, and BiOI sample was attained by a similar method without NCC, CN_2_H_4_S and acid solutions, as well.

### Characterizations

The crystal structure, surface element state, micro-morphology, optical properties, specific surface area, and aperture analysis of samples were studied by XRD (AXS D8 ADVANCE), XPS (ESCALAB 250Xi, Thermo Fisher Scientific), SEM, TEM (Tecnai G2 F30 S-Twin), UV-Vis DRS, and BET (ASAP 2460, Micromeritics) technics, respectively.

### Photocatalytic Activity Testing

The photocatalytic reactivity testing was carried out by measuring the degradation of 20 mg L^−1^ rhodamine B (RhB) aqueous solution. A 500 W Xe lamp (λ > 420 nm) was used as a light source. For each experiment, 30 mg of photocatalytic material including the NCC support was used. In the beginning, the photocatalysts and RhB mixture was stirred for 1 h in dark to reach the adsorption-desorption equilibrium. The adsorption efficiency of RhB in darkness was texted by the control experiment: the concentration of the dark adsorbed solution was compared with 20 mg L^−1^ RhB aqueous solution for 2 h by recording the adsorption intensity via a UV-Vis spectrophotometer. While under the light, 3 mL solution was withdrawn every 20 min and the concentration was tested by recording the adsorption intensity via a UV-Vis spectrophotometer.

### Electrochemical Testing

Electrochemical impedance spectroscopy (EIS) was conducted using a CHI660D electrochemical workstation in a standard three-electrode system with a Pt counter electrode, Ag/AgCl (saturated KCl) reference electrode, and nickel foam as a working electrode. The working electrodes were prepared as follows: 30 mg of photocatalyst powder was dispersed in 1 mL of absolute ethyl alcohol with 50 μL polytetrafluoroethylene by sonication for 30 min, then the obtained slurry was dip-coated on the nickel foam. The electrolyte was a 0.2 mol L^−1^ Na_2_SO_4_ aqueous solution. The working electrode was dried and soaked in the electrolyte for 6 h. Electrochemical impedance spectroscopy (EIS) was recorded at frequency ranged from 0.01 to 10^5^ Hz with an AC voltage magnitude of 5 mV.

## Results and Discussion

### Phase Structures

**Figure 1:**
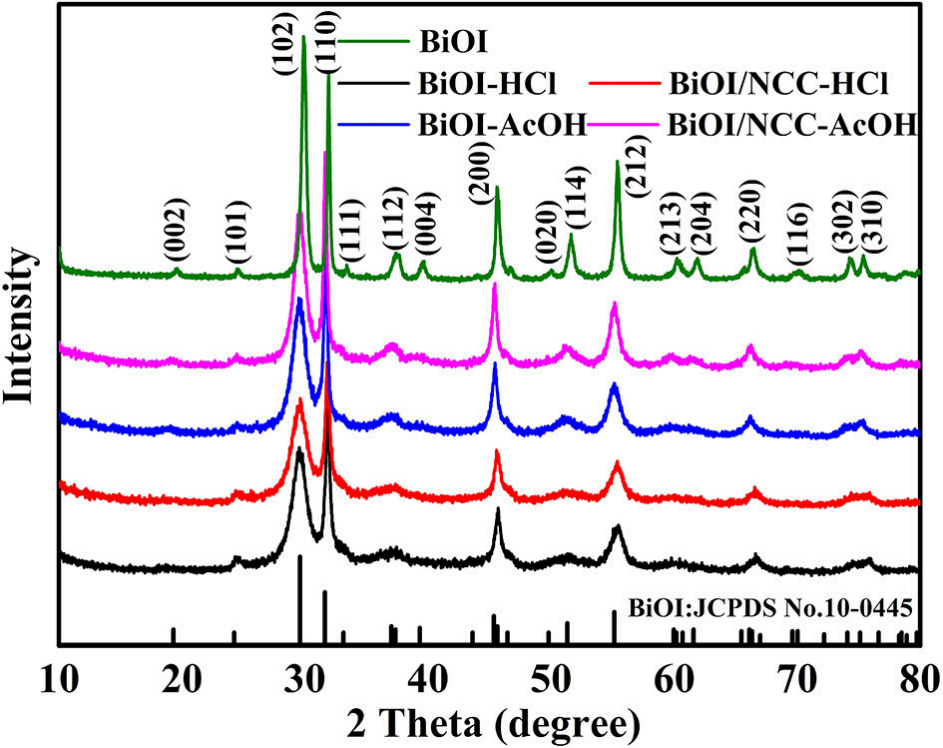
XRD patterns of BiOI, BiOI-HCl, BiOI-AcOH, BiOI/NCC-HCl, and BiOI/NCC-AcOH.

The crystalline structure was studied by XRD (Figure 1). The diffraction peak of BiOI sample was well matched with the standard card number JCPDS 10-0445, indicating pure BiOI material with distinct crystallinity (Hou et al., 2018). Besides, for the XRD patterns of BiOI-HCl, BiOI-AcOH, BiOI/NCC-HCl, and BiOI/NCC-AcOH, no new diffraction peaks were observed due to use of different acids (HCl and AcOH). However, in comparison to the crystal structure of pure BiOI, the diffraction peaks of BiOI-HCl, BiOI-AcOH, BiOI/NCC-HCl, and BiOI/NCC-AcOH are broader and weaker, representing that their grain size is smaller than BiOI and their crystallinity is also reduced. Moreover, for BiOI/NCC-HCl and BiOI/NCC-AcOH XRD patterns, no significant change was also observed. Moreover, compared with pure BiOI, the peak at around 29.2° displayed a tiny shift toward the left side after HCl treatment, which may due to the formation of new material from the surface of pure BiOI reacts with an acid solution. In addition, the diffraction peaks of carbon were not appeared in the XRD patterns of the BiOI/NCC-HCl and BiOI/NCC-AcOH samples, due to a small amount of NCC.

### Chemical States

**Figure 2:**
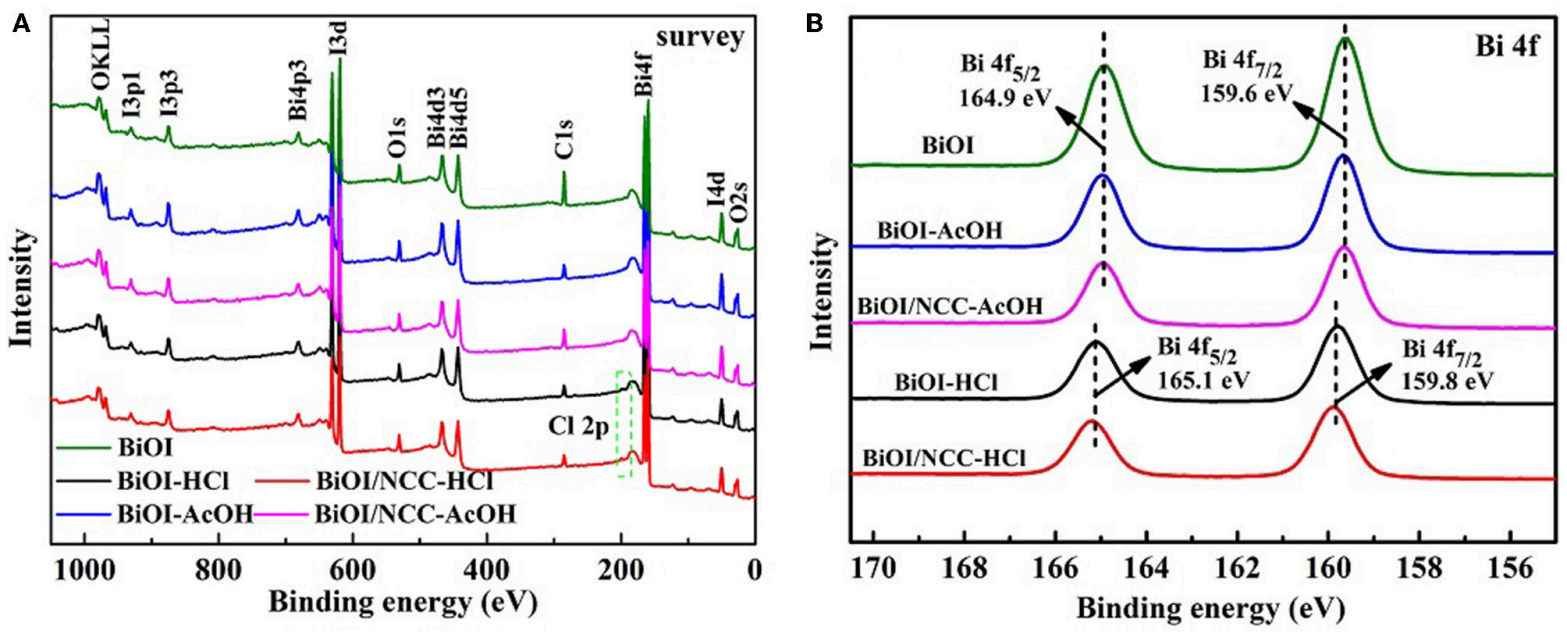
XPS analysis of **(A)** full survey and **(B)** A Bi 4f spectra of BiOI, BiOI-HCl, BiOI-AcOH, BiOI/NCC-HCl, and BiOI/NCC-AcOH.

XPS analysis (Figure 2) was performed to test the surface element state. The full survey spectra (Figure 2A) showed that all the five samples contained Bi, O, and I elements. Moreover, the distribution proportions of elements are listed in Table S1. The XPS survey spectrum in Figure 2A demonstrated C, O, Bi, and I elements in the all sample, while BiOI-HCl materials contained a small amount of Cl elements. XPS result showed the content ratio of Bi, I, and Cl elements are 1:0.89:0.10, which was close to EDX result of 1:0.85:0.09 of pure BiOI treated with HCl. When HCl solution was added to the pure BiOI, the surfaces of BiOI were dissolved by acid, Cl^−^ gradually replaces I^−^, to form BiOI_0.9_Cl_0.1_. However, after treatment of acetic acid solution, the content ratio of Bi and I elements were all close to 1:0.98 by XPS result of BiOI-AcOH, which was close to EDX result of 1: 0.96. From the Bi 4f spectra (Figure 2B), the binding energies of BiOI sample at 159.6 and 164.9 eV stand for Bi 4f_7/2_ and Bi 4f_5/2_ of Bi^3+^ ions, respectively, and the same with BiOI-AcOH and BiOI/NCC-AcOH samples, indicating that the treatment with AcOH cannot change the chemical environment of Bi^3+^ ions of BiOI. While treated with HCl, the Bi 4f_7/2_ and Bi 4f_5/2_ peaks of BiOI-HCl and BiOI/NCC-HCl displayed 0.2 eV shift toward higher binding energy, showing a higher charge of Bi ions, which may due to the formation of BiOI_0.9_Cl_0.1_. The generation of BiOI_0.9_Cl_0.1_/NCC heterojunction with multiple charge transfer channels is beneficial to improve the separation of photoinduced electron-hole pairs for efficient photocatalytic degradation (Deng et al., 2018).

### Surface Morphology Structure

**Figure 3:**
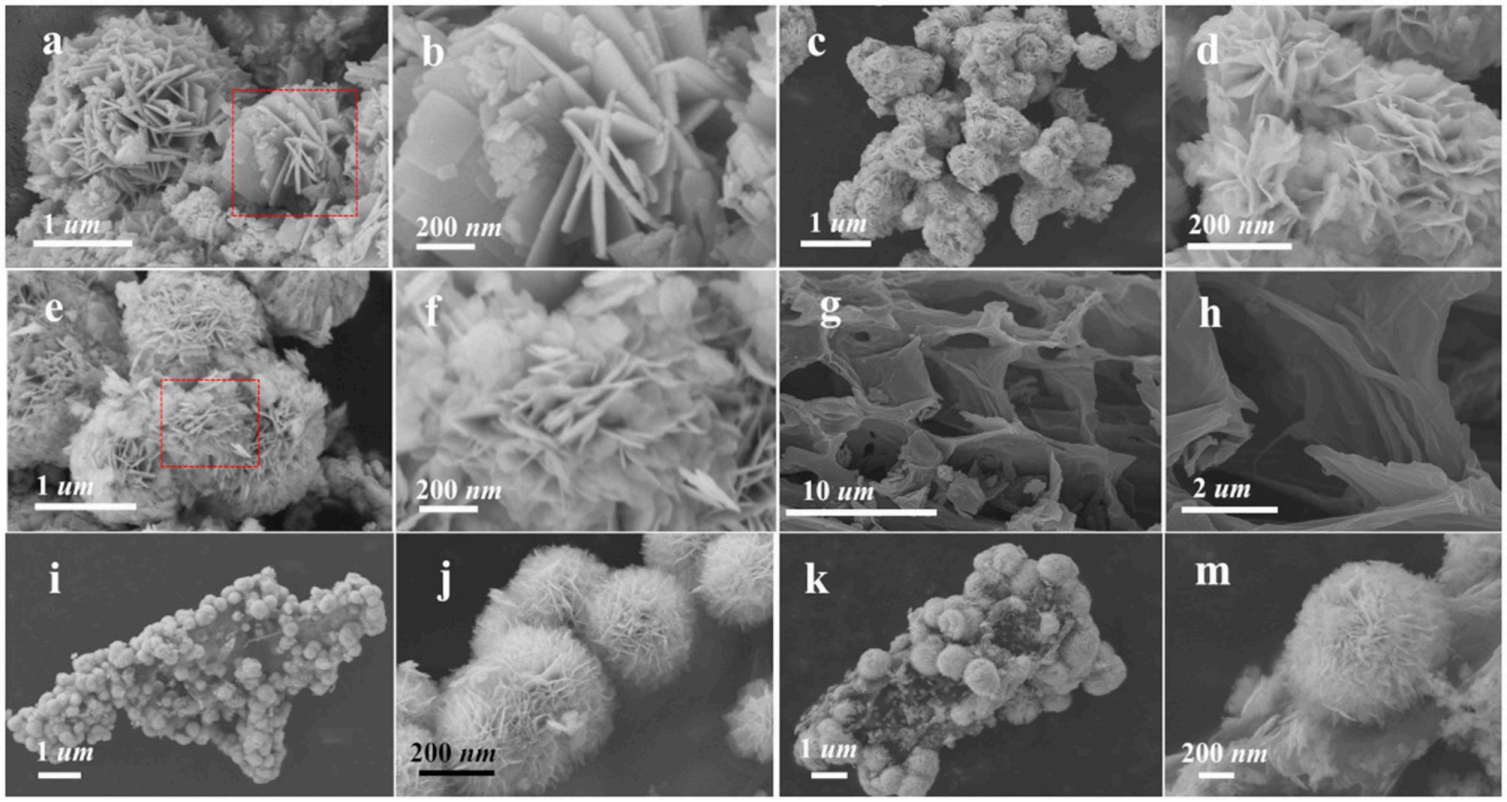
SEM images of **(a,b)** BiOI, **(c,d)** BiOI-HCl, **(e,f)** BiOI-AcOH, **(g,h)** NCC, **(i,j)** BiOI/NCC-HCl, and **(k,m)** BiOI/NCC-AcOH.

**Figure 4:**
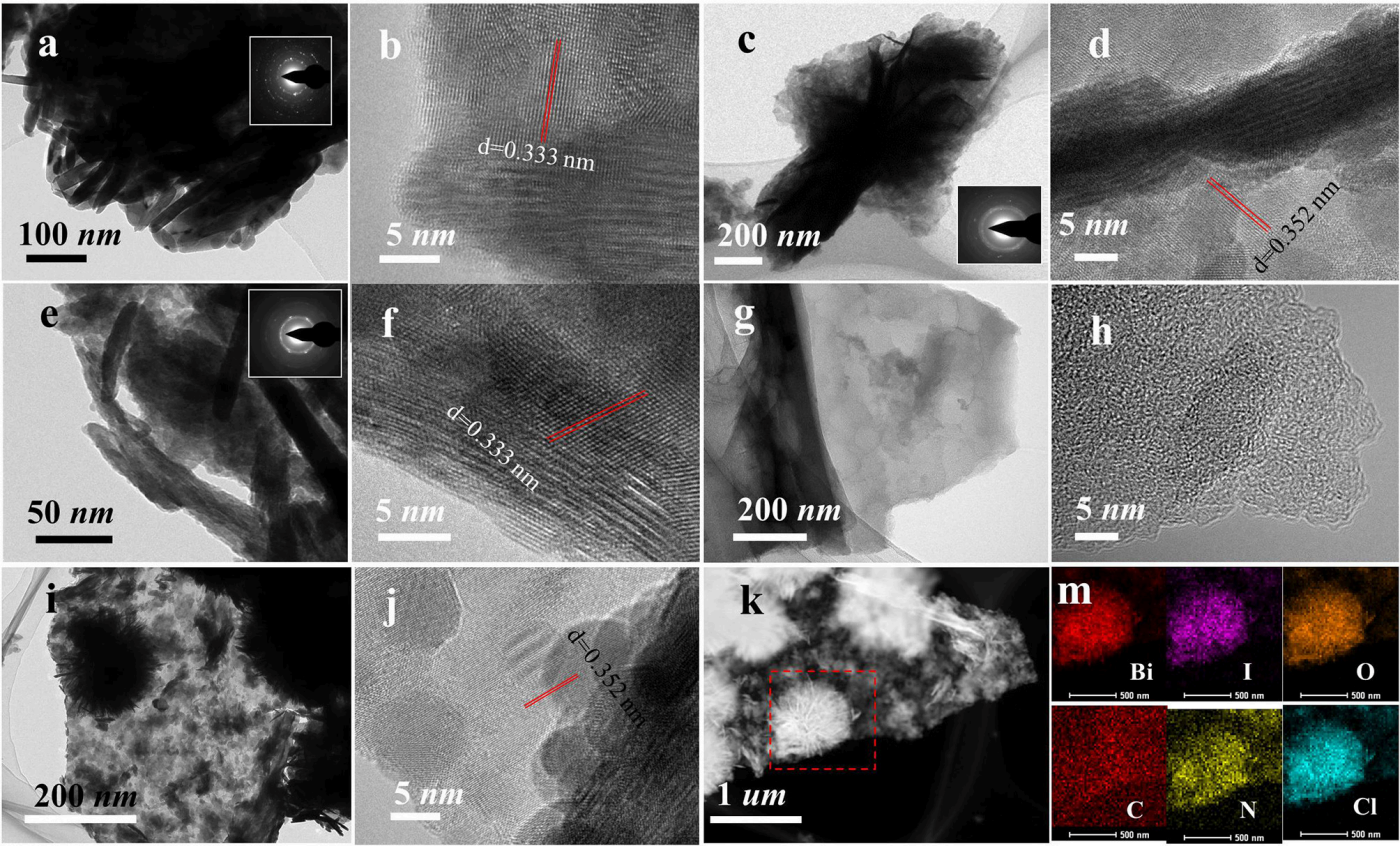
TEM images of **(a,b)** BiOI, **(c,d)** BiOI-HCl, **(e,f)** BiOI-AcOH, **(g,h)** NCC, **(i,j)** BiOI/NCC-HCl, and **(k,m)** EDX elemental mapping of BiOI/NCC-HCl.

Morphological analysis of as-prepared material was done by using SEM (Figure 3) and TEM (Figure 4). It is shown in Figures 3a,b, BiOI sample reveals many disordered sheet structures stacked together with a smooth surface. While after treating with acidic solutions (HCl and AcOH), the obtained BiOI-HCl (Figures 3c,d) and BiOI-AcOH (Figures 3e,f) converted to microsphere structures composed of agglomerated nanosheets. These nanosheets become thinner due to the assistant dissolve effect of acidic solutions (Ding et al., 2015), which helped to shorten the diffusion path to reduce the photo-generated carrier recombination rate (Zhang et al., 2014; Huang et al., 2016; Jing et al., 2016; Jiang et al., 2017). Reconnection of the tubular structure of N-doped cattail-based carbon material (NCC) obtained through the direct carbonization of cattail grass. NCC obtained with high specific surface areas, which provided enough contact area to BiOI materials. The formed characteristic hierarchical cellular structure provided the loading capacity for active molecules, ions, and/or even micron-sized materials (Deng et al., 2018), especially the aperture of macropores (>2 μm) is larger than BiOI microsphere (< 1 μm), which provided enough load space for the BiOI microspheres to load onto the NCC as shown in Figures 3i–m. With the NCC as carriers, the strong electronegativity of N atoms on the surface attracted the Bi cations providing the preferential nucleation sites (Hou et al., 2018; Lu et al., 2018; Yin et al., 2019). Consequently, BiOI microspheres were formed at the nucleation sites on NCC through the auto self-assembly of BiOI nanosheets (Wang H. et al., 2018). By adding acidic solutions, BiOI/NCC material acted as the self-sacrifice template, following the assistant dissolve effect of acid (Ding et al., 2015). In the result, the BiOI/NCC-HCl (Figures 3i,j) and BiOI/NCC-AcOH (Figures 3k,m) with well-defined hierarchical ultrathin nanosheets structure were obtained, which shorten the diffusion pathways of pollutants and greatly expand the efficiency of photogenerated charge carrier's diffusion to the surface (Jing et al., 2016; Sun et al., 2018). From Figures 3i,k, the surface of NCC is tightly packed with many homogeneous BiOI microspheres composed of ultrathin nanoparticles, which provided sufficient interfacial interaction between BiOI and NCC (Hou et al., 2018). During the photocatalytic process, NCC worked as an electron-scavenger and transfer photogenerated electrons rapidly through the interfacial effect between NCC and BiOI to obtain effective separation of photoelectrons and holes, which facilitated the more effective redox reaction (Hou et al., 2015, 2017, 2018; Jing et al., 2016; Ye et al., 2017; Di et al., 2018; Lu et al., 2018; Yin et al., 2019).

From Figures 4a,b, the TEM images of BiOI sample showed the existence of a laminar structure, and the SAED pattern (the illustration in Figure 4a) directs the polycrystalline phase structure that could aggregate easily. The TEM images of BiOI-HCl (Figures 4c,d) and BiOI-AcOH (Figures 4e,f) after the treatment with HCl and AcOH also demonstrated the lamellar structures. Nonetheless, the SAED patterns of BiOI-HCl (Figure 4c) and BiOI-AcOH (Figure 4e) indicated that they are all polycrystalline. As shown in Figures 4g,h, after 750°C carbonized process in ammonia gas, the obtained NCC retained the intrinsic framework of cattail grass. The product demonstrated a unique distribution of interconnected hierarchy, which could provide ideal load capacity for semiconductor materials such as BiOI (Hou et al., 2015, 2017, 2018; Jing et al., 2016; Di et al., 2018; Lu et al., 2018; Yin et al., 2019). After NCC was combined with BiOI and treated with HCl, it could be observed from the TEM pattern of BiOI/NCC-HCl (Figures 4i,j) that BiOI microspheres were grown over NCC and the ultrathin slices of the microspheres which are visibly clear. The EDX elemental mapping of BiOI/NCC-HCl (Figure 4m) showed that Bi, I, O, N, and Cl elements were homogeneously distributed over C element, where N elements appeared due to N-doping in NCC.

At the well-contacted interface between NCC and BiOI, NCC could serve as an electron-acceptor bridge for ultrafast electron transfer during photocatalysis process (Hou et al., 2018). Moreover, Cl-doping generation of BiOI/BiOCl/NCC heterojunctions provided multiple charge transfer channels beneficial to improve the separation of photoinduced electrons and holes for efficient photocatalytic degradation. (Deng et al., 2018) In addition, BiOI-HCl (Figure 4d) and BiOI/NCC-HCl (Figure 4j) have a plane spacing of ~0.352 nm, significantly greater than BiOI and BiOI-AcOH samples (~0.333 nm). Considering that Cl is a much smaller anion than I, Cl^−^ gradually replace the original position of I^−^ in the process of synthesizing BiOI_0.9_Cl_0.1_, which makes the distance between the neighboring atoms larger.

**Figure 5:**
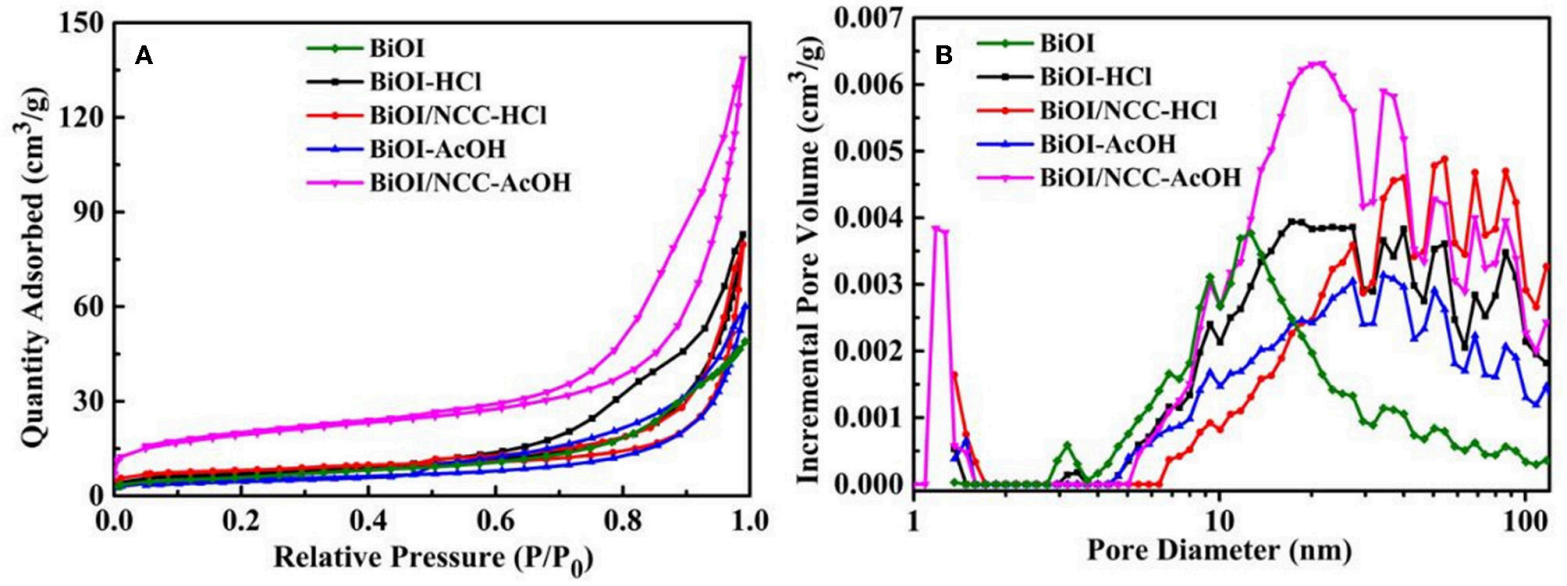
**(A)** Nitrogen adsorption-desorption isotherms and **(B)** corresponding pore size distributions of BiOI, BiOI-HCl, BiOI-AcOH, BiOI/NCC-HCl, and BiOI/NCC-AcOH.

Figure 5 showed the nitrogen adsorption-desorption isotherms and corresponding pore size distributions of the samples, specific surface area and porosity properties which were summarized in Table S2. The specific surface areas of BiOI-HCl, BiOI-AcOH, BiOI/NCC-HCl, and BiOI/NCC-AcOH are 24.7, 28.5, 27.2, and 36.3 m^2^ g^−1^, respectively, all are higher than that of pure BiOI (21.5 m^2^ g^−1^). As higher surface area can provide more active sites, as well as wider interfacial interactions, enabling efficient electrons-transfer from BiOI to the absorbed pollutant or NCC at the material surface (Hou et al., 2018). From Figure 5B, the pore diameter of BiOI sample was distributed in mesopore and macropore, without microporous and its average pore diameter was 13.8 nm. While with the treatment of two acidic solutions, the pore sizes of BiOI-HCl and BiOI-AcOH samples were also mainly distributed in mesopore and macropore, and the average pore diameters were 20.7 and 20.1 nm, respectively. However, by combining with NCC, the obtained BiOI/NCC-HCl and BiOI/NCC-AcOH samples presented the extra micropores of 1–1.8 nm, which may result from the defects formed by NCC and contacted interface between NCC and BiOI. In addition, BiOI/NCC-HCl, and BiOI/NCC-AcOH have much higher volumes of the mesopore-macropore network than the other three samples because of the hierarchical structure between the interconnected ultrathin nanosheets in BiOI microspheres and NCC, which was beneficial to the rapid degradation of pollutants (Zhang et al., 2014; Huang et al., 2016; Jing et al., 2016; Jiang et al., 2017).

### Optical and Electrical Properties

After the treatment of acid, the BiOI-HCl and BiOI-AcOH exhibited slightly blue-shift. The reason is that an acidic treatment has developed ultrathin structures with a wider band gap, consistent with previous reports (Di et al., 2016; Jiang et al., 2017). But the degree of red-shift was obviously increased after combining with the carbon material. This narrowing should be attributed to the chemical bonding between BiOI and NCC, which is the formation of Bi-O-C band (Liu et al., 2013). For the BiOI/NCC-HCl and BiOI/NCC-AcOH, the composition of NCC broaden up the absorption range, which increased the visible light utilization rate of BiOI/NCC composite materials.

**Figure 6:**
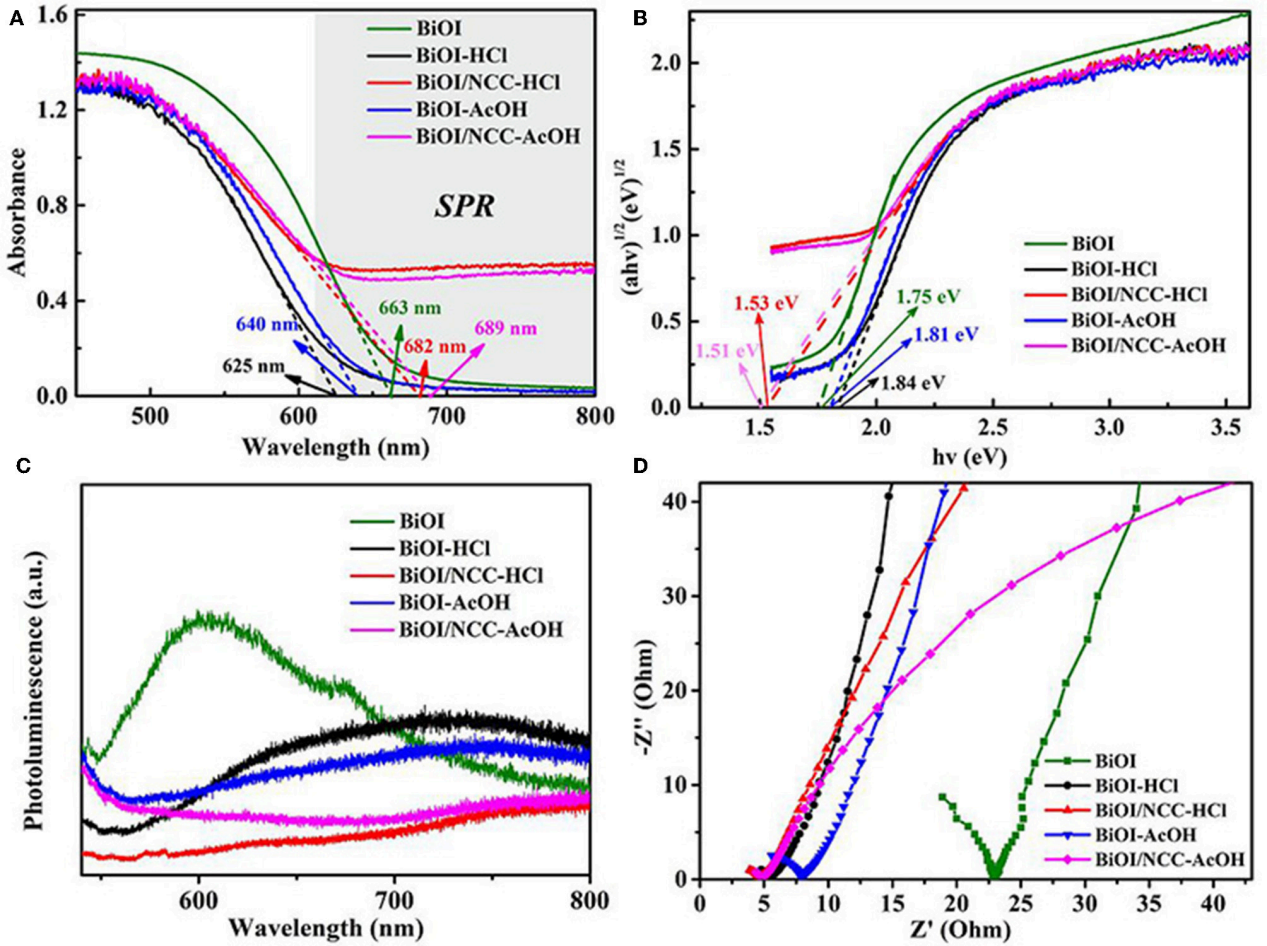
**(A)** UV-Vis diffuse reflectance spectra, **(B)** plots of (αhv)^1/2^ vs. *hv*, **(C)** photoluminescence spectra, and **(D)** Electrochemical impedance spectroscopy (EIS) of BiOI, BiOI-HCl, BiOI-AcOH, BiOI/NCC-HCl, and BiOI/NCC-AcOH samples.

From Figure 6A, the maximum absorption wavelength of BiOI, BiOI-HCl, BiOI-AcOH, BiOI/NCC-HCl, and BiOI/NCC-AcOH samples are 663, 625, 640, 682, and 689 nm, respectively. The introduction of conductive NCC could enhance the excitation and transfer of the surface plasmon resonance (SPR) hot electrons, which could trigger the generation of reactive species with high oxidation capacity, leading to superior photocatalytic efficiency for environmental remediation (Ma et al., 2016; Liu W. et al., 2017; Zhang et al., 2018). Moreover, as shown in Figure 6B by using the formula: α*hv* = *A* (*hv*-*E*_*g*_)^*n*/2^, where α, ν, E_g_, and A represent the absorption coefficient, light frequency, band gap energy, and a constant, respectively (Liu et al., 2015; Di et al., 2016; Jiang et al., 2017; Wang Q. et al., 2018; Zhang et al., 2018). For BiOX materials, *n* is 4 for its indirect transition. Then the band gap energies of the samples were assessed using a plot of (α*hv*)^1/2^ vs. photon energy (*hv*). And the bandgap E_g_ of BiOI, BiOI-HCl, BiOI-AcOH, BiOI/NCC-HCl, and BiOI/NCC-AcOH estimated are 1.75, 1.84, 1.81, 1.53, and 1.51 eV, respectively. After the treatment of acid, the BiOI-HCl and BiOI-AcOH exhibited slightly blue-shift, the reasons may be that an acidic treatment developed ultrathin structures that can extend the band gap, consistent with previous reports (Di et al., 2016; Jiang et al., 2017). But the degree of red-shift was obviously increased after combining with carbon material. This narrowing should be attributed to the chemical bonding between BiOI and NCC, which is the formation of Bi-O-C band (Liu et al., 2013). For the BiOI/NCC-HCl and BiOI/NCC-AcOH, the composition of NCC broaden up the absorption range, which can increase the visible light utilization rate of BiOI/NCC composite materials. Figure 6C shows the photoluminescence (PL) spectra of all samples. Compared to BiOI, BiOI-HCl, BiOI/NCC-HCl, BiOI-AcOH, and BiOI/NCC-AcOH have a much lower peak intensity of PL due to the modification of acid treated and NCC. A weaker intensity of the PL suggested a lower efficiency of electron-hole recombination. Figure 6D shows EIS of all samples revealing semicircle arcs and straight lines. Taking the same preparation of electrodes and electrolyte into account, the high-frequency semicircles can be attributed to the resistance of the electrodes. As compared to pure BiOI, the impedance plot of BiOI-HCl and BiOI-AcOH exhibit smaller radius, respectively, BiOI/NCC-HCl and BiOI/NCC-AcOH exhibit the smallest radius, respectively, showing faster interfacial electron transfer. Therefore, the addition of NCC will facilitate the interfacial charge transfer by acting as an electron-acceptor, which inhibits the recombination of photoinduced electron-hole pairs.

### Photocatalytic Activity Testing

**Figure 7:**
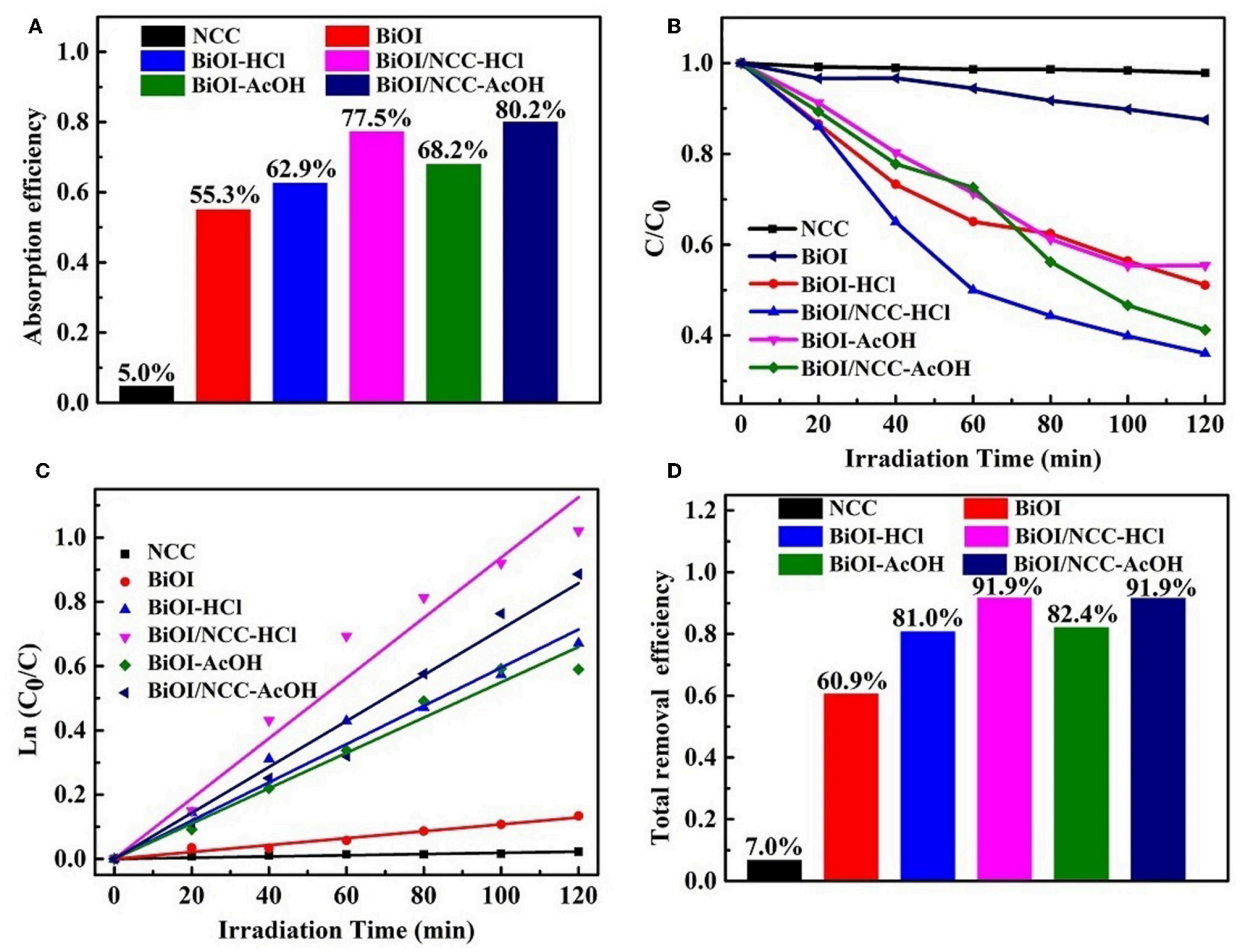
**(A)** Adsorption efficiency of RhB in darkness, **(B)** photocatalytic degradation of RhB, **(C)** plot of ln (C_0_/C) vs. irradiation time during photocatalytic degradation, and **(D)** total removal efficiency of RhB for the BiOI, BiOI-HCl, BiOI-AcOH, BiOI/NCC-HCl, and BiOI/NCC-AcOH samples.

The photocatalytic activity was performed through the photo-degradation of organic dye (rhodamine B, RhB) under simulated visible light (Figure 7). The adsorption efficiency (Figure 7A) of RhB in darkness for NCC, BiOI, BiOI-HCl, BiOI-AcOH, BiOI/NCC-HCl, and BiOI/NCC-AcOH samples were 5.0, 55.3, 62.9, 68.2, 77.5, and 80.2%, respectively. This showed both the acid treatment and the combination of NCC can extend the adsorption capacity. It is obvious from Figure 7B that both acid treatment and recombination of NCC can improve the photocatalytic efficiency, and thus BiOI/NCC-HCl and BiOI/NCC-AcOH can degrade organic pollutants 4~5 times more than pure BiOI under simulated visible light irradiation. Figure 7C is the study of photocatalytic degradation kinetics of the pollutant RhB. Using the following formula: ln (C_0_/C) = kt, the corresponding first-order rate constant k was obtained. After measurement, the rate constant k of NCC, BiOI, BiOI-HCl, BiOI-AcOH, BiOI/NCC-HCl, and BiOI/NCC-AcOH were 0.00018, 0.00108, 0.00595, 0.0055, 0.00938, and 0.00715 min^−1^, BiOI/NCC-HCl and BiOI/NCC-AcOH responded with the fastest degradation reaction rate than pure BiOI (nine times faster). After dark adsorption and simulation of photocatalytic degradation, the total removal efficiency of RhB by NCC, BiOI, BiOI-HCl, BiOI-AcOH, BiOI/NCC-HCl, and BiOI/NCC-AcOH are 7.0, 60.9, 81.0, 82.4, 91.9, and 91.9%, respectively (Figure 7D). The photocatalytic experiments show that NCC nearly cannot degrade RhB, but the addition of NCC enhance the photocatalytic activity of BiOI/NCC samples compared with BiOI samples, which further prove that the composition of NCC broadens up the absorption range, which can increase the visible light utilization rate of BiOI/NCC composite materials.

Thus, the above experiments indicated that the combination with NCC and acid treatment can cause higher degradation efficiency and faster degradation speed for a simulated pollutant (RhB), especially BiOI/NCC-HCl, compared with other samples. This is largely due to the following points: (1) the ultrathin-layer structure can cause a faster transfer of the photoinduced charge carriers by shortening the transmission pathways, which is in favor of access to surface catalysis sites easily (Zhang et al., 2014; Huang et al., 2016; Jing et al., 2016; Jiang et al., 2017); (2) the micro/nano hierarchical three-dimensional microsphere structures can facilitate the shorten diffusion pathways of pollutants and can also greatly promote the diffusion of photoinduced charge carriers to the surface (Hou et al., 2015, 2018); (3) high specific surface area can provide multiple active sites (Hou et al., 2017); (4) N-doped hierarchical cattail-based carbon (NCC) can serve as an electron scavenger, thus can speed up the separation of photoelectrons and holes through well-contacted interface (Hou et al., 2015, 2017, 2018; Jing et al., 2016; Di et al., 2018; Lu et al., 2018; Yin et al., 2019); (5) NCC functioned as a conductive support, which can transfer the electrons and enhance the SPR effect toward efficient generation of reactive species for environmental applications; (6) The Cl element is critical for the generation of BiOI/BiOCl/NCC heterojunction, which is beneficial to improve the separation of photoinduced electrons and holes for efficient photocatalytic degradation (Liu W. et al., 2017). From the above, this work utilized a one-step, green, and facile method to prepare BiOI/NCC composites with better photocatalytic performance and systematically investigated the *in-situ* self-template and its contribution to RhB degradation. Therefore, our work can provide a new dimension toward BiOI-based photocatalysts for high-performance visible photocatalysis.

## Conclusion

In summary, self-assembled BiOI microspheres with ultrathin-layer structure loaded on biochar (N-doped cattail-based carbon, NCC), namely BiOI/NCC composite materials were produced through one-step, green and facile approach by the *in-situ* self-template. In the synthesis process, acidic solutions (HCl or AcOH) served as a solvent to acquire ultrathin nanosheets, and NCC acted as a conductive carrier for the formation of BiOI microspheres. The as-synthesized micro/nano hierarchical NCC can provide plenty of surface interfaces with even-distributed BiOI microspheres composed of ultrathin nanosheets, which were generated on the surface and inside of NCC. Owing to the multiple synergistic effects of micro/nano hierarchical microsphere structure, ultrathin nanosheets, and well-contacted interface, the BiOI/NCC composites exhibited the excellent photocatalytic activity for degradation (RhB) than pure BiOI under visible light. Consequently, BiOI/NCC-HCl showed the most superb photocatalytic performance owing to the presence of Cl element, and the developed BiOI/BiOCl/NCC heterojunction also improved the separation of photoinduced electrons and holes. Thus, our work demonstrated the facile and controlled synthesis routes which could provide an effective method to design and optimized BiOX (X = halides) based photocatalytic materials.

## Author Contributions

The original idea was generated by JH. All lab experiments were performed by JH and compilation of properties measurements were also done by him. FI helped with writing up the results and compiling the results together. The final modifications were done by DB. TJ and RW prepared the samples and performed the experiments.

### Conflict of Interest Statement

The authors declare that the research was conducted in the absence of any commercial or financial relationships that could be construed as a potential conflict of interest.
